# Environmental endocrine disruptors and endometrial cancer: a systematic review of epidemiological studies

**DOI:** 10.3389/fendo.2026.1719784

**Published:** 2026-02-12

**Authors:** Dalila Incognito, Claudia Gelsomino, Antonio Picone, Giuseppe Ettore, Carla Ettore, Giosuè Giordano Incognito, Giordana Di Mauro, Mariacarmela Santarpia, M’Hammed Aguennouz, Silvana Parisi, Giuliana Ciappina, Massimiliano Berretta

**Affiliations:** 1 Medical Oncology Unit, Department of Human Pathology “G. Barresi”, School of Specialization in Medical Oncology, University of Messina, Messina, Italy; 2 Translational Molecular Medicine and Surgery, Department of Clinical and Experimental Medicine, University of Messina, Messina, Italy; 3 Department of Clinical and Experimental Medicine, University of Messina, Messina, Italy; 4 Obstetrics and Gynecology Unit, Maternal Child Department, ARNAS Garibaldi Nesima, Catania, Italy; 5 Department of Human Pathology “G. Barresi”, Medical Oncology Unit, University of Messina, Messina, Italy; 6 Radiation Oncology Unit, Department of Biomedical, Dental Science, and Morphological and Functional Images, University of Messina, Messina, Italy; 7 Department of Medical Sciences, Section of Experimental Medicine, University of Ferrara, Ferrara, Italy

**Keywords:** bisphenol A, cadmium, endometrial cancer, environmental exposure, phytoestrogens

## Abstract

Endometrial cancer (EC) is a hormone-dependent malignancy whose global incidence is steadily rising. Increasing evidence suggests that exposure to endocrine-disrupting chemicals (EDCs) may contribute to EC development through interference with hormonal signaling mediated by estrogen receptors (ER). This systematic review aimed to synthesize the available epidemiological evidence on the association between exposure to EDCs and the risk of EC. A comprehensive search of MEDLINE, EMBASE, Scopus, the Cochrane Library, and ClinicalTrials.gov was conducted up to April 2025, following PRISMA 2020 recommendations. Eligible studies included observational or interventional designs assessing quantified exposure to EDCs and EC incidence or risk. Eight studies met the inclusion criteria (five case-control, two cohort, and one randomized controlled trial), comprising 2, 609 EC cases and 1, 577 controls. Cadmium emerged as the EDC most frequently associated with EC, reported in five studies, four of which demonstrated a significant positive association, particularly among lean postmenopausal women. In contrast, evidence regarding bisphenol A (BPA) and its metabolites was inconsistent: only mono-n-butyl phthalate (MnBP) showed a positive correlation with EC in one study, while BPA and dibutyl phthalate (DBP) did not. Nonylphenol and octylphenol were also found to be positively associated with EC risk in a single study. Conversely, neither lead exposure nor dietary intake of isoflavones was associated with EC incidence. Overall, available epidemiological evidence supports a potential association between cadmium exposure and increased EC risk, while data for other EDCs remain limited and inconclusive. Substantial heterogeneity in exposure assessment, study design, and confounder adjustment limits comparability across studies and precludes definitive conclusions. Further well-designed longitudinal studies with standardized and repeated exposure measurements are needed to clarify causal relationships between EDC exposure and endometrial carcinogenesis.

**Systematic Review Registration:**

https://inplasy.com/inplasy-2025-6-0030/, identifier INPLASY202560030.

## 1 Introduction

Endometrial cancer (EC) is the sixth most frequently diagnosed malignancy in women, with approximately 417, 000 new cases worldwide and nearly 97, 000 deaths reported each year (1, 2). It is classified historically into two main histopathological subtypes. Type I tumors, which account for about 85% of cases, are predominantly low-grade endometrioid carcinomas characterized by a glandular growth pattern, low histological grade, and high expression of estrogen receptor (ER)-α, indicating a hormone-driven pathogenesis. In contrast, Type II tumors include high-grade endometrioid carcinomas, serous, clear cell, and carcinosarcomas, as well as mixed histology tumors, which exhibit more aggressive behavior and are less dependent on hormonal signaling (3–6). This traditional dichotomous model has been partially superseded by a molecular classification that better captures the biological heterogeneity of EC, identifying distinct subgroups with specific prognostic and therapeutic implications, including POLE-ultramutated, mismatch repair-deficient, p53-abnormal, and tumors with no specific molecular profile (7). Within this evolving molecular framework, increasing attention has been directed toward environmental and exogenous factors that modulate hormonal and intracellular pathways involved in endometrial carcinogenesis. Among these, endocrine-disrupting chemicals (EDCs) represent a heterogeneous class of compounds that interfere with hormonal regulation and have been increasingly implicated in the development of hormone-dependent malignancies, including EC (8, 9). Some EDCs share structural similarities with endogenous steroid hormones, such as estradiol (E2), enabling them to bind to hormone receptors and potentially modulate estrogen-dependent signaling pathways that are critical in EC development (10). EDCs include persistent contaminants such as dioxins, polychlorinated biphenyls, and brominated flame retardants, as well as pesticides including triazoles, dicarboximides, and triazines. Industrial compounds such as phthalates and bisphenol A (BPA), along with natural substances like phytoestrogens, are also recognized as EDCs (10). Experimental studies have demonstrated that selected EDCs can activate ER dependent transcription, induce oxidative stress, and promote proliferative and anti-apoptotic signaling pathways that are relevant to endometrial carcinogenesis (3). However, EDCs represent a biologically diverse group, and their effects on endometrial tissue are not uniform (3). Preliminary studies have shown that synthetic compounds, such as BPA, phthalates, and heavy metals like cadmium, may exhibit estrogenic activity, induce oxidative stress, promote cellular proliferation, and inhibit apoptosis (8, 9). In contrast, naturally occurring substances such as phytoestrogens, including isoflavones and lignans, may have protective effects due to their antioxidant, anti-inflammatory, and anti-proliferative properties (11, 12). Nevertheless, their estrogenic activity and selective ER modulator-like behavior indicate that prolonged exposure or high-dose supplementation may, under specific conditions, stimulate endometrial proliferation and have been associated with endometrial hyperplasia in clinical and experimental studies (13). These plant-derived molecules can act as selective estrogen receptor modulators (SERMs), potentially mitigating the harmful estrogenic effects of other EDCs (11). Despite the growing body of mechanistic evidence, epidemiological data linking EDC exposure to EC risk remain inconsistent and fragmented, and no prior synthesis has systematically focused on human studies addressing this association. Given the heterogeneity of available data, a systematic and critical evaluation of the existing epidemiological evidence is required to clarify the association between EDCs and EC risk. The present review aims to assess human studies investigating the association between EDC exposure and EC risk, integrating epidemiological findings with relevant mechanistic insights to identify areas of consistency, sources of discrepancy, and key knowledge gaps that should be addressed in future research.

## 2 Materials and methods

A systematic review was performed in accordance with the Preferred Reporting Items for Systematic Reviews and Meta-Analyses (PRISMA) guidelines (14). The protocol was retrospectively registered on INPLASY (number: INPLASY202560030, registration date: June 7, 2025). The included studies evaluated the association between exposure to EDCs and EC. A comprehensive bibliographic search was conducted, covering all relevant literature from inception to April 2025. Databases searched included Medline, Embase, Scopus, the Cochrane Database of Systematic Reviews, and ClinicalTrials.gov. The following search terms were used: “endometrial cancer” (MeSH Unique ID: D016889), “endocrine disruptors” (MeSH Unique ID: D052244), “bisphenol A Compounds” (MeSH Unique ID: D000098826), “cadmium” (MeSH Unique ID: D002104), “dibutyl phthalate” (MeSH Unique ID: D003993), “phytoestrogens” (MeSH Unique ID: D010784), “isoflavones” (MeSH Unique ID: D020862), and “lignans” (MeSH Unique ID: D008096). All search terms were combined using Boolean operators (AND/OR), with “endometrial cancer” used in combination with each EDC-related term to ensure retrieval of studies specifically addressing the association between EDC exposure and EC. The search was restricted to human studies in English language. Commentaries, letters to the editors, editorials, reviews, and conference abstracts were excluded. Eligibility criteria were defined using the population, intervention, comparator, outcomes and study design (PICOS) framework, as summarized in **Table 1**.

**Table 1:** Eligibility criteria structured according to the PICOS framework.

Component	Inclusion criteria	Exclusion criteria
Population	Women with a histologically confirmed diagnosis of EC (cases) and women without EC (controls or non-cases, including participants with benign uterine conditions).	Animal or *in vitro* studies.
Intervention	Quantified EDCs exposure.	No EDCs exposure assessment.
Comparison	Studies comparing women across exposure levels, using those with lower or background exposure as reference.	Studies without internal exposure-level comparison.
Outcomes of interest	EC incidence or risk.	No EC outcomes.
Designs	Observational studies (cohort, case-control) and RCTs reporting data on EC incidence or risk in relation to.	Review articles, meta-analyses, editorials, commentaries, letters, conference abstracts.

EC, endometrial cancer; EDCs, endocrine-disrupting chemicals; NA, not available; RCTs, randomized controlled trials

The population of interest included women of any age or menopausal status. Cases were defined as women with histologically confirmed EC, regardless of exposure level. Controls were women without EC, including healthy participants or those with benign uterine conditions, as specified in each study. Exposure to EDCs represented the primary independent variable and was quantified through biomonitoring (urine, blood, or plasma) or validated dietary and environmental assessment methods. The timing of exposure assessment relative to EC diagnosis was extracted and classified as prospective (prediagnostic), cross-sectional (at diagnosis), or postdiagnostic (after histological confirmation but before treatment), as summarized in **Table 2**. The association between exposure level and EC occurrence was evaluated by comparing biomarker concentrations or estimated exposure across cases and controls. Comparators were defined by lower or background levels of exposure within the same population. The outcome of interest was the incidence or risk of EC associated with different levels of exposure to EDCs. In cohort studies, EC incidence referred to the number of new EC cases identified during the observation period among women with varying degrees of EDC exposure. In case-control studies, EC risk was defined as the relative likelihood of having EC in relation to exposure level, expressed as odds ratios or adjusted relative risks derived from biomarker or dietary exposure data. Eligible study designs included observational studies (case-control and cohort) and randomized controlled trials (RCTs) providing quantitative exposure outcome data. Studies involving animals, *in vitro* models, or outcomes unrelated to EC were excluded.

**Table 2:** Characteristics of the studies included in the systematic review.

Author, year	Study type	Sample size	Cases	Controls	Mean age (years)	Mean BMI (kg/m^2^)	EDCs	Timing of exposure assessment relative to EC diagnosis	Exposure assessment	Association with EC risk
Sarink et al., 2021 (15)	Case-control	139 cases vs 139 controls	Post-menopausal women with EC	Post menopausal women without EC	Cases: 62 Controls: 62	Cases: 28 Controls: 26.3	BPA, MnBP, DBP	6.6 years between sample collection and diagnosis	Urine	MnBP, DBP: positive (2nd tertile only); BPA: null
McElroy et al., 2017 (16)	Case-control	631 cases vs 879 controls	Women with EC	Women without EC	Cases: 60.1 Controls: 62.9	Cases: 37.5 Controls: 29.3	Cadmium	Cross-sectional (diagnosis at sampling)	Urine	Positive
Michalczyk et al., 2023 (17)	Case-control	21 cases vs 89 controls	Women with EC	Women with other uterine pathologies or normal endometrium; 25 patients with uterine myomas; 18 patients with normal endometrium; 48 patients with endometrial polyps	Cases: 70 Polyps: 51	Cases: 30.27 Polyps: 26.46 Normal endometrium: 29.72 Myomas: 25.28	Cadmium, lead	Cross-sectional (postdiagnostic blood sampling before surgery)	Plasma	Cd: positive; Pb: null
Wen et al., 2020 (18)	Case-control	49 cases vs 101 controls	Women with EC	Women without EC	Cases: 50.94 Controls: 44.79	Cases: 26.35 Controls: 23.06	NP, OP	Cross-sectional	Urine	Positive
Akesson et al., 2008 (19)	Prospective cohort	378 cases vs 30 controls	Women with EC	Women without EC	Cases: 60.1 Controls: 62.9	25.3-25.4	Cadmium	Prospective (prediagnostic dietary exposure assessment)	Dietary intake	Positive
Eriksen et al., 2014 (20)	Prospective cohort	192 cases vs 24 controls	Women with EC	Women without EC	Cases: 57 Controls: 57	26	Cadmium	Prospective (prediagnostic dietary exposure assessment)	Dietary intake	Positive in women with BMI <25 kg/m^2^
Quaas et al., 2013 (21)	RCT	1 case of EC in the placebo group vs 224 controls divided into two groups (103 in the placebo group, 121 in the isoflavone soy protein group)	Stage IB EC diagnosed in the placebo group after an initial finding of complex endometrial hyperplasia with atypia	Post menopausal women without endometrial hyperplasia or cancer	56	25	Soy isoflavones	Prospective (3-year intervention period)	Plasma	Null
Adams et al., 2014 (22)	Prospective cohort	1.198 cases vs 91 controls	Women with EC	Women without EC	62	28	Cadmium	Prospective (prediagnostic dietary exposure assessment)	Dietary intake	Null

BMI, body mass index; BPA, bisphenol A; Cd, cadmium; DBP, dibutyl phthalate; EC, endometrial cancer; EDCs, endocrine-disrupting chemicals; MnBP, mono-n-butyl phthalate; Pb, lead; RCT, randomized clinical trial; y, years.

**Figure 1:**
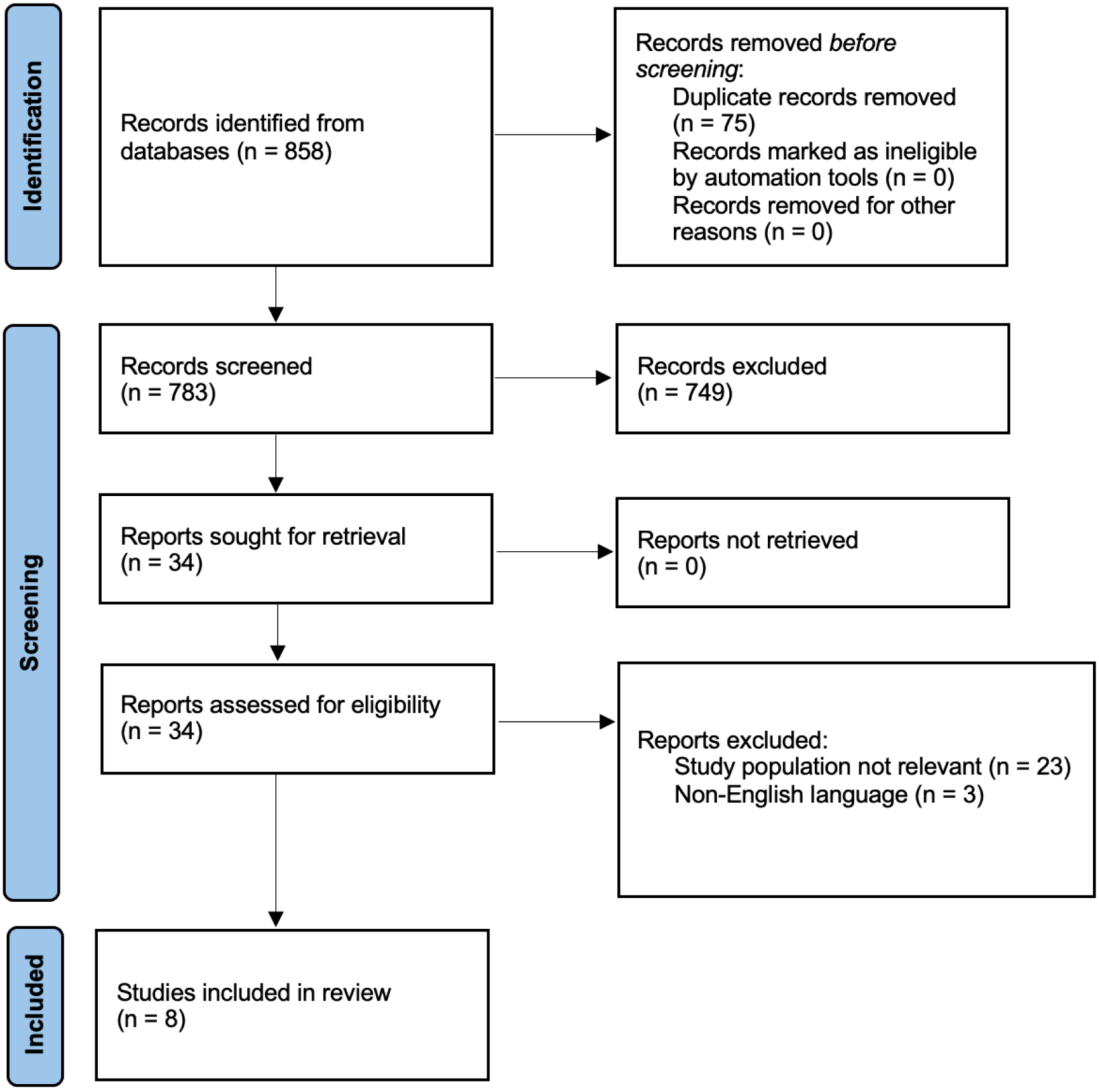
PRISMA flow diagram.

**Figure 2:**
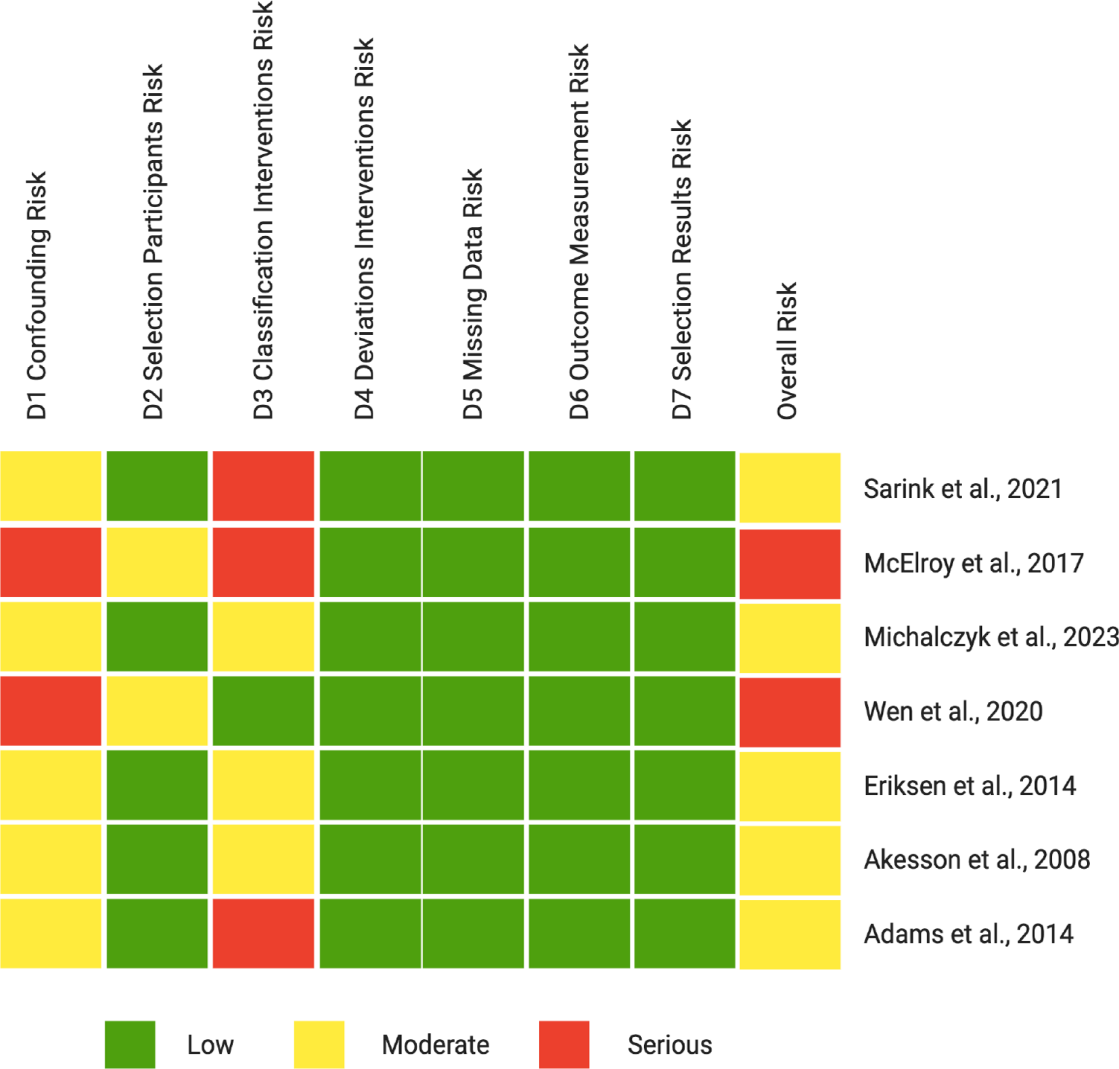
Risk of bias heatmap for observational studies using the ROBINS-I tool.

Search results were imported into Qatar Computing Research Institute (QCRI) Rayyan software for systematic screening (23). Two authors (D.I. and C.G.) independently reviewed the titles and abstracts of each citation for relevance and selected articles for full-text review. Discrepancies were resolved by consensus or discussion with a third investigator (M.B.). A PRISMA flow diagram was created to summarize the selection process (**Figure 1**). Two authors (D.I. and C.G.) independently extracted the data from each eligible study using a standardized electronic form. Any discrepancies were resolved by consensus or, if necessary, through consultation with a third author (M.B.). For each study, the following information were collected: author and year of publication, country, study design, sample size (cases and controls), population characteristics (age, body mass index - BMI, and any relevant inclusion or exclusion criteria), type of EDCs assessed, timing of exposure assessment relative to EC diagnosis, and biological matrix used for exposure assessment, outcomes of interest (EC incidence or risk). The methodological quality and risk of bias of the included studies were assessed independently by two authors (C.E. and G.G.I.) using the ROBINS-I tool (24) for observational studies and the RoB 2 tool (25) for RCTs. Each domain was critically examined to ensure consistency and transparency of evaluation. Specifically, the ROBINS-I tool assessed seven domains: bias due to confounding, bias in participant selection, bias in exposure classification, bias due to deviations from intended exposures, bias due to missing data, bias in outcome measurement, and bias in selection of the reported results. The RoB 2 tool evaluated bias arising from the randomization process, deviations from intended interventions, missing outcome data, outcome measurement, and selection of the reported result. Discrepancies between reviewers were resolved through discussion and consensus with a senior investigator (M.B.). Each domain was rated as low, moderate, serious, or critical risk of bias, and an overall judgment was assigned to each study in accordance with PRISMA 2020 recommendations. The evaluation also accounted for major confounding factors, including age, BMI, menopausal status, smoking, and hormone therapy use, which were systematically extracted and considered in the interpretation of results. The statistical robustness of each study was assessed based on whether adjusted risk estimates, confidence intervals, and multivariable models were reported. Risk of bias plots were generated using RStudio (version 4.5.0) and the robvi package, summarizing the distribution of bias across domains (**Figure 2**). Due to the heterogeneity of study designs, exposure assessment methods, and outcome definitions, a quantitative meta-analysis was not performed. Findings were synthesized narratively, with particular attention to methodological quality, consistency of associations, and exposure metrics.

## 3 Results

**Figure 1** summarizes the process of identifying and selecting the relevant literature studies.

A systematic bibliographic search identified 858 records. At the title and abstract screening stage, 749 records were excluded because they did not meet the predefined inclusion criteria, mainly due to the absence of a direct assessment of EDCs exposure, lack of EC-specific outcomes, non-human study design, or inappropriate study type (e.g., reviews, editorials, or case reports). During the screening process, 749 records were excluded. Subsequently, 34 full-text articles were assessed for eligibility; 23 studies were excluded due to a non-relevant population, and 3 studies were in a non-English language. Ultimately, 8 studies met the inclusion criteria and were included in the systematic review (15–22) (**Table 2**).

The selected studies, published between 2008 and 2023, included both case-control and prospective cohort designs, and included a total of 2, 609 EC cases and 1, 577 controls. The mean age of women diagnosed with EC was reported in five studies (15, 17–20) and ranged from 50.94 (18) to 70 years (17). The mean BMI, available in four studies (16, 18, 21, 22), ranged from 25 (21) to 37.5 kg/m² (16) in patients with EC. In several studies, age and BMI were reported only in aggregate form or were not stratified by case/control status.

Cadmium was the most frequently investigated EDCs evaluated in 5 out of 8 studies (16, 17, 19, 20, 22). Four of these studies (80%) reported a positive association between cadmium exposure and EC risk (16, 17, 19, 20), while one study did not identify a statistically significant association (22).In some studies, this association appeared more pronounced in specific subgroups, such as women with a body mass index below 25 kg/m² (15, 17). BPA, MnBP, and DBP were assessed in a single nested case-control study (15). A positive association was reported for MnBP, while BPA and DBP showed no consistent link with EC risk. Another study evaluated urinary exposure to nonylphenol (NP) and octylphenol (OP), reporting a positive association between elevated urinary levels and increased risk of EC (18). One study presented findings from a RCT on long-term isoflavone soy supplementation, reporting no increase in EC risk: a single case was documented in the placebo group, with no cases observed in the intervention group (21).

The results of the risk of bias assessment are summarized in **Table 3**.

**Table 3:** Risk of bias assessment for the included studies.

Study	Study design	Tool	Risk of bias assessment
Sarink et al., 2021 (15)	Case-control	ROBINS-I	Moderate: single urine sample; confounders adjusted
McElroy et al., 2017 (16)	Case-control	ROBINS-I	Low: large sample, biomonitoring, confounders well controlled
Michalczyk et al., 2023 (17)	Case-control	ROBINS-I	Moderate: biomonitoring data; limited sample size
Wen et al., 2020 (18)	Case-control	ROBINS-I	Serious: small sample, one time measurement, limited confounder control
Eriksen et al., 2014 (20)	Prospective cohort	ROBINS-I	Moderate: self-reported exposure data; stratified analysis reduces some bias
Akesson et al., 2008 (19)	Prospective cohort	ROBINS-I	Low: long term follow up, dietary cadmium assessed, confounding addresse
Quaas et al., 2013 (21)	RCT	RoB 2	Low: proper randomization, blinding, low attrition
Adams et al., 2014 (22)	Prospective cohort	ROBINS-I	Moderate: dietary cadmium estimated by model; residual confounding likely

RCT: Randomized Controlled Trial; ROBINS-I: risk of bias in non-randomized studies of interventions; RoB 2: Cochrane risk of bias 2 tool for randomized trials.

Subgroup patterns related to BMI were identified; however, they were limited by incomplete stratification in the primary studies. Sensitivity analyses could not be performed due to the small number of studies and the heterogeneity in exposure assessment and outcome reporting.

**Figure 3:**
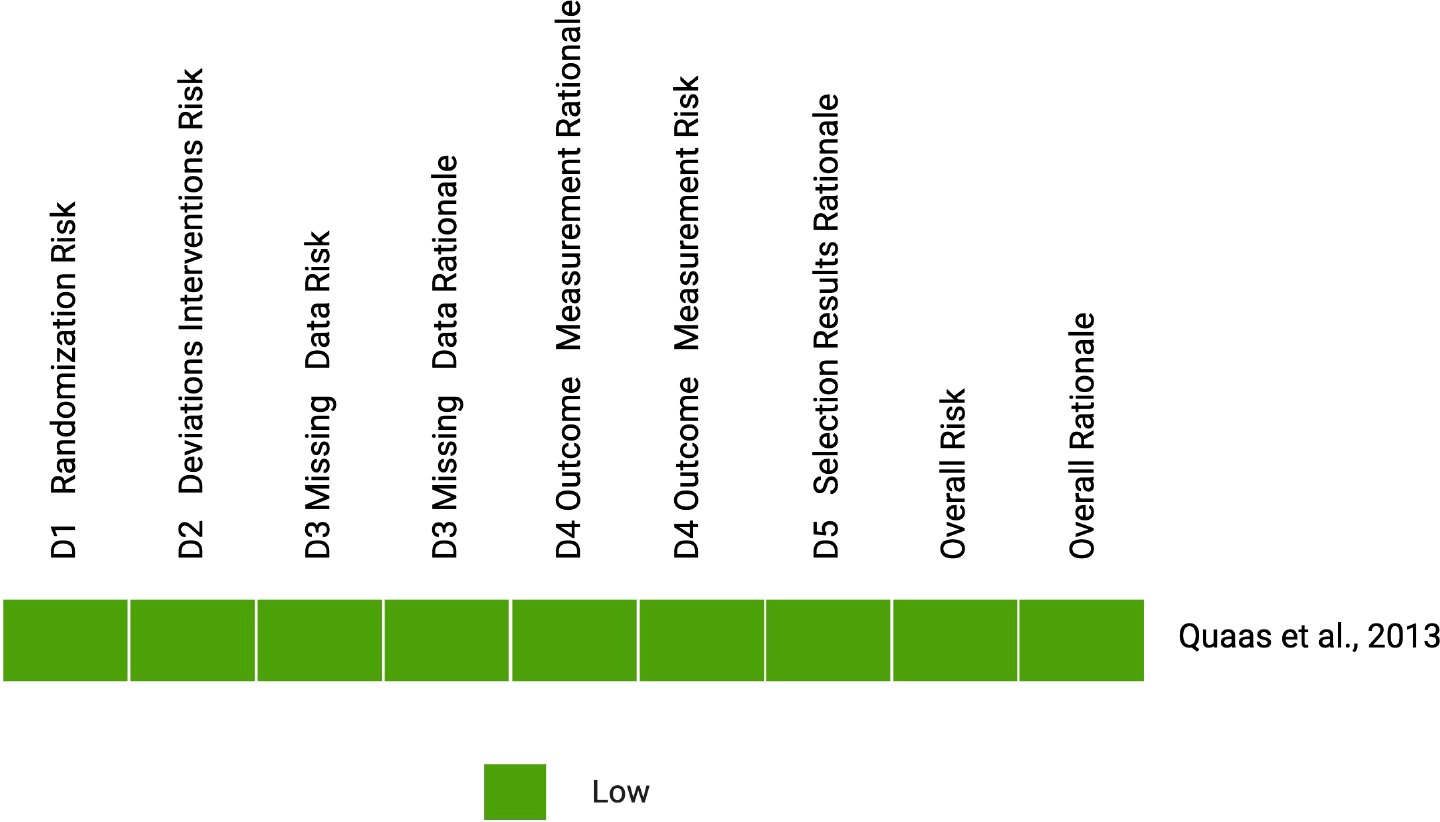
Risk of bias assessment for the randomized controlled trial using the RoB 2 tool.

The results of the risk of bias assessments are presented in **Figure 2** (ROBINS-I) and **Figure 3** (RoB 2).

## 4 Discussion

### 4.1 Summary of the main findings

**Figure 4:**
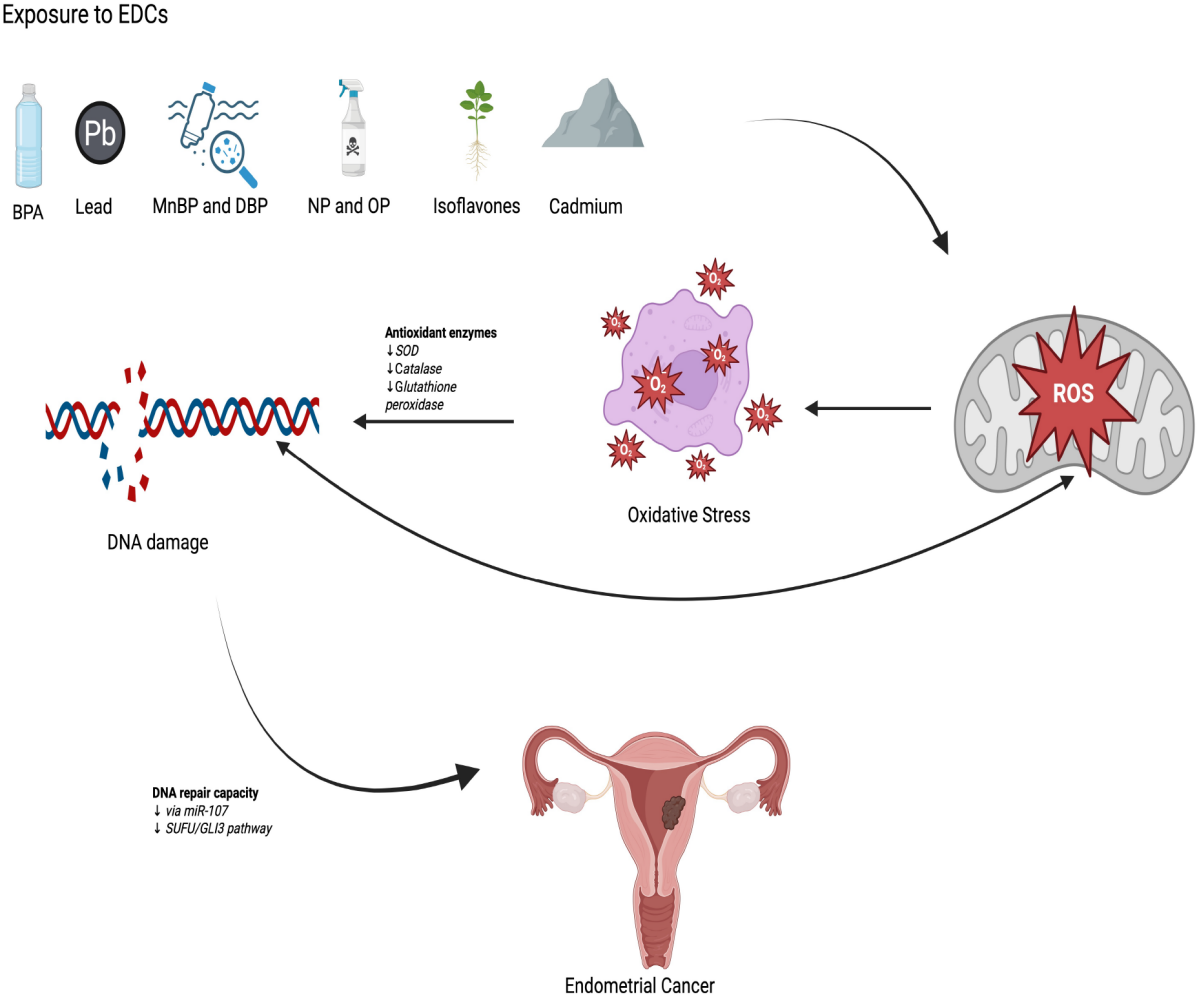
Pathogenetic model linking exposure to EDCs with EC through oxidative stress and DNA damage. ROS can interact directly with DNA, causing structural modifications of the nitrogenous bases. ROS can also oxidize other cellular biomolecules, such as lipids and proteins, generating reactive intermediates that can subsequently interact with DNA, resulting in indirect genotoxic effects. Persistent oxidative stress may overwhelm cellular defense mechanisms, leading to DNA damage accumulation and contributing to endometrial carcinogenesis.

This systematic review evaluated the relationship between exposure to EDCs and the risk of EC in eight human studies, including both case-control and prospective cohort designs. Cadmium was the most frequently investigated compound, analyzed in five studies, four of which reported a positive association with EC risk, particularly among women with lower body mass index. BPA and the phthalates MnBP and DBP were assessed in one nested case-control study, in which a positive association was observed only for MnBP. NP and OP were evaluated in a single study, in which higher urinary concentrations were linked to an increased EC risk. Lead was examined together with cadmium and was not significantly associated with EC risk. One RCTinvestigated long-term isoflavone supplementation and was not significantly associated with EC risk. Taken together, the epidemiological evidence identified in this review is limited in number and heterogeneous in design and exposure assessment. However, the relatively consistent signal observed for cadmium, compared with other EDCs, supports a potential association with EC risk. These findings gain further relevance when interpreted in light of experimental data indicating that selected EDCs may influence estrogen-related signaling, oxidative stress pathways, and epigenetic regulation in endometrial tissue.A schematic overview of the oxidative stress-related mechanisms linking EDC exposure to endometrial carcinogenesis is provided in **Figure 4**.

### 4.2 Bisphenol A

BPA has attracted particular attention in EC research due to its widespread human exposure and potential estrogenic activity. BPA is a synthetic compound that is commonly used in the production of plastics and various consumer products (26, 27). It is also a key component in the production of epoxy resins, which are used to create coatings and protective films for food and beverage containers (28, 29). Chronic low-dose exposure may occur through dietary intake as a result of BPA leaching from food contact materials (28). Although the effects of BPA on women’s health have been extensively investigated, its specific role in EC remains incompletely defined. Experimental studies indicate that BPA can interfere with multiple cellular processes relevant to endometrial carcinogenesis, including cell cycle regulation, DNA damage response, migration, and invasion (30–33). It has been shown to promote cell proliferation, epithelial-to-mesenchymal transition, and increase COX-2 expression, thereby contributing to enhanced migratory and invasive properties in EC cells (14, 10, 34–36). BPA can induce oxidative stress, leading to mitochondrial dysfunction, lipid peroxidation, DNA damage, and the activation of pro-inflammatory pathways, such as NF-κB, which may promote tumorigenesis (27, 32). At the molecular level, BPA exerts estrogen-mimetic effects through interactions with ER signaling (35). Experimental data demonstrate that BPA can bind to ERs, alter gene expression, and activate signaling pathways, such as MAPK1, AKT1 and PIK3CA, contributing to endometrial cell proliferation through multiple mechanisms (35, 36). Experimental data have further demonstrated that BPA acts as a xenoestrogen by binding to both nuclear ERα and ERβ and the membrane-associated G protein-coupled ER (GPER), leading to activation of downstream signaling cascades such as PI3K/AKT and MAPK/ERK, which promote proliferation and inhibit apoptosis in endometrial epithelial cells (37).

*In vitro* studies in human endometrial adenocarcinoma cell lines have shown that BPA upregulates COX-2 and VEGF expression via activation of ERK and NF-κB pathways, supporting its role in inflammatory and angiogenic signaling relevant to tumor progression (38).

Furthermore, BPA exposure has been shown to induce mitochondrial dysfunction and increase reactive oxygen species (ROS) production, leading to oxidative DNA damage and impaired repair responses (37, 38).

Epigenetic alterations have also been reported, including aberrant DNA methylation and modulation of microRNAs such as miR-149 and miR-107, which regulate genes involved in cell cycle control and DNA damage response (30, 31). The structural similarity of BPA to E2 allows it to mimic endogenous estrogen and directly bind to ERα, ERβ, and GPER, thereby reproducing the receptor-mediated transcriptional activation typical of estrogen-dependent endometrial proliferation. Together, these findings support a mechanistic framework in which BPA may promote estrogen-responsive proliferation, oxidative stress, inflammation, and epigenetic dysregulation in endometrial cells (39, 40). However, current epidemiological evidence does not support these experimental observations. A case-control survey by Sarink et al. (15) examined the association between exposure to EDCs and the onset of EC, analyzing urine samples collected prospectively several years before diagnosis. In this study involving 139 postmenopausal women, urinary BPA excretion levels were comparable between EC cases and controls. The majority of cases presented with endometrioid histology (75%) and localized disease (71%). One limitation of this study was the reliance on a single urine sample. Accordingly, despite substantial biological plausibility derived from experimental models, available human data do not demonstrate a significant association between BPA exposure and EC risk.

### 4.3 Dibutyl phthalate, mono-n-butyl phthalate

Phthalates (MnBP and DBP) have also been investigated for their potential role in endometrial carcinogenesis. These compounds are released from plastic products and are classified as EDCs due to their ability to alter endocrine system function (9, 41). Dietary intake represents one of the main exposure routes in the general population. In experimental and translational models, phthalates have been shown to influence cell proliferation and tumor-related pathways in estrogen-responsive tissues, including the endometrium (41, 42). Experimental evidence suggests that DBP and its metabolite MnBP may affect endometrial cells through a combination of estrogenic, oxidative, and epigenetic mechanisms (42, 43). Specifically, phthalates can promote ROS generation and suppress antioxidant defense systems, including glutathione peroxidase and catalase. This imbalance leads to mitochondrial dysfunction, lipid peroxidation, and DNA damage (40–43). Beyond oxidative stress, DBP and MnBP act by binding to ERα and peroxisome proliferator-activated receptor-γ (PPARγ), thereby interfering with endogenous estrogenic signaling and modulating the transcription of genes involved in cell proliferation and differentiation (42). Furthermore, these compounds induce epigenetic alterations, including DNA hypomethylation and histone acetylation changes, which further impair normal endometrial differentiation and affect tumor-suppressor gene regulation (42, 43). Together, these mechanisms create a pro-oxidant and estrogen-responsive environment that can facilitate malignant transformation in hormone-dependent endometrial tissue (42).

In the case-control study by Sarink et al. (15), the authors did not indicate a clear linear increase in risk for any of the EDCs analyzed. Specifically, for MnBP, the association was significant only in the second tertile compared to the first. The absence of a consistent trend across higher exposure categories limits the interpretability and strength of this finding. Similar patterns were observed for total DBP metabolites, with a significant association restricted to the second tertile. Overall, the lack of a monotonic dose-response relationship precludes firm conclusions regarding a causal association between phthalate exposure and EC risk and highlights the need for further investigation.

### 4.4 Cadmium

Cadmium is a hazardous metal naturally present in the environment and released as a pollutant from industrial and agricultural activities (44–46). In non-smokers, food represents the primary source (46). Various factors, including nutritional status, such as iron deficiency influence its bioavailability, accumulation, and toxicity. Cadmium is primarily stored in the kidneys, where it has a long half-life of 10–30 years, and its levels correspond to those detected in urine (41–43). It is classified by the International Agency for Research on Cancer (IARC) as a Group 1 human carcinogen. Its carcinogenic effects in the endometrium are thought to involve two main pathways: oxidative stress and estrogen mimicry (26). First, cadmium promotes ROS generation and interferes with antioxidant defense systems, such as glutathione and catalase, resulting in oxidative DNA lesions, chromosomal aberrations, and impaired DNA repair (26, 47). Second, it acts as a metalloestrogen, directly activating ERα and GPER. This activation stimulates downstream PI3K/AKT and MAPK/ERK signaling, upregulates estrogen-responsive genes including c-fos and cyclin D1, and leads to increased proliferation and inhibition of apoptosis in endometrial cells (48, 49). Furthermore, cadmium has been shown to induce hypermethylation of tumor suppressor genes, reinforcing its dual oxidative and estrogenic carcinogenic potential (48). Given the established role of unopposed estrogen as a central risk factor for EC, this malignancy represents a particularly informative model for evaluating these metalloestrogenic effects (50). In the population-based case–control study by McElroy et al. (16), urinary cadmium levels were measured cross-sectionally at the time of diagnosis. The authors found that higher cadmium exposure was significantly associated with an increased risk of EC (OR: 1.22; 95% CI: 1.03–1.44). Michalczyk et al. (17) analyzed whole blood samples collected after histological diagnosis but before surgical treatment. Their data show that cadmium is a risk factor for EC. Higher blood cadmium levels were significantly associated with an increased likelihood of an EC diagnosis (OR = 5.25; 95% CI: 1.56–17.72). The risk was also elevated in older women (OR = 8.28; 95% CI: 2.20–31.16) and those who were postmenopausal (OR = 14.77; 95% CI: 1.86–117.17). These findings support the hypothesis that cumulative cadmium exposure may exert more substantial carcinogenic effects in hormonal or metabolic contexts characterized by reduced estrogen clearance or increased tissue susceptibility (51).In a prospective cohort study within the large population-based Swedish Mammography Cohort, Akesson et al. (19) assessed dietary cadmium exposure at baseline and during a 10-year follow-up period. Their findings suggest a statistically significant association, with an even more substantial effect observed in women with low levels of both endogenous and exogenous estrogen exposure. Conversely, Adams et al. (22) conducted a large prospective cohort analysis within the Women’s Health Initiative and reported no statistically significant association between dietary cadmium intake and EC risk after adjusting for potential confounders. Similarly, Eriksen et al. (20) evaluated dietary cadmium exposure prospectively in postmenopausal women enrolled in the Diet, Cancer and Health Study. They reported a positive association only in women with a BMI <25 kg/m^2^, while no association was observed in those with a BMI ≥25 kg/m^2^. Overall, while cadmium shows the most consistent associations with EC risk among the EDCs reviewed, the discrepancies likely reflect differences in exposure assessment methods (urinary, blood, or dietary), timing of exposure measurement, and effect modification by hormonal and metabolic factors such as menopausal status and BMI (51). Future studies integrating precise exposure metrics with molecular and hormonal profiling are needed to identify susceptible subgroups more accurately.

### 4.5 Lead

Lead has been less extensively studied in relation to EC, but its potential carcinogenicity merits attention. Elevated concentrations have been associated with an increased risk of various cancers, including those of the lung, kidney, gastrointestinal tract, breast, and gynecological regions. The IARC has classified inorganic lead and lead compounds as probably carcinogenic to humans (41). Its carcinogenic mechanisms are thought to involve oxidative stress, interaction with zinc finger proteins, induction of apoptosis, and disruption of cell signaling pathways (52, 53). Sources of lead exposure include food, water, gasoline, contaminated dust, and direct contact with contaminated materials. ROS can interact directly with DNA, causing structural modifications of the nitrogenous bases. ROS can also oxidize other cellular biomolecules, such as lipids and proteins, generating reactive intermediates that can subsequently interact with DNA, resulting in indirect genotoxic effects (54, 55). In experimental models, lead exposure has been shown to modulate estrogen-related pathways through indirect mechanisms rather than direct receptor binding (52, 54). Recent mechanistic studies indicate that lead exposure can activate ERα and upregulate matrix metalloproteinases (MMP-2 and MMP-9) through ROS-dependent NF-κB signaling, promoting cell proliferation and invasion in uterine and ovarian models, suggesting a potential estrogen-mimetic effect relevant to endometrial tissue (56). Notably, available evidence suggests that these estrogen-like effects are mediated by redox-dependent modulation of intracellular signaling cascades that converge on estrogen-responsive gene expression, rather than by direct high-affinity binding to ER (10). Lead exposure has also been shown to enhance oxidative stress and impair mitochondrial function, leading to lipid peroxidation, DNA damage, and activation of NF-κB and MAPK pathways. In this context, lead has been described as a weak xenoestrogen, reflecting its ability to indirectly potentiate estrogen responsive signaling without acting as a classical ER agonist (49). However, Michalczyk et al. (17) found no significant associations between blood lead levels, the cadmium-to-lead ratio, and EC risk in their case-control study. This lack of epidemiological association suggests that the estrogen mimetic effects observed in experimental systems may be insufficient, on their own, to promote endometrial carcinogenesis at environmentally relevant exposure levels (57). Accordingly, current human data do not support a major role for lead exposure in EC development, although the limited number of available studies highlights a gap in current knowledge and underscores the need for further investigation (57).

### 4.6 Nonylphenol and octylphenol

NP and OP are alkylphenols that are primarily used as intermediates in the chemical manufacturing industry. NP is also used in applications such as labeling tax-favored light fuel oil, as a preservative agent, in the tanning industry, and in pesticide formulations (55, 58). Both compounds are classified as EDCs due to their estrogen like activity and widespread environmental presence (58). One possible mechanism linking NP and OP exposure to EC development is the activation of the pregnane X receptor (PXR) (58). NP and OP also exert estrogen-like activity and have been shown in animal models to induce endometrial hyperplasia and increase uterine weight, mimicking the effects of E2. Mechanistically, NP-mediated PXR activation has been shown to upregulate CYP3A enzymes involved in steroid hormone metabolism, potentially altering estrogen homeostasis and contributing to oxidative imbalance, DNA damage, and activation of carcinogenic pathways (59). However, these effects have been primarily observed in experimental systems and are not specific to endometrial tissue. Epidemiological evidence is limited to a single case-control study by Wen et al. (18), which reported a positive association between urinary NP and OP concentrations and EC risk. Interpretation of these findings is constrained by methodological limitations, including reliance on a single urine sample for exposure assessment. Given the short biological half-life of NP and OP, estimated at approximately 2–3 hours in blood (60, 61), single-sample measurements may not accurately reflect long-term exposure. Accordingly, current human data should be considered preliminary, and the available evidence is insufficient to draw firm conclusions regarding a causal association between NP, OP exposure, and EC risk. Confirmation in larger studies with repeated exposure measurements is warranted.

### 4.7 Isoflavones

Isoflavones are a type of phytoestrogen, naturally occurring nonsteroidal phenolic plant compounds that, due to their molecular structure and size, resemble vertebrate steroid estrogens (60–62). Many women use isoflavone soy protein (ISP) supplements as an alternative to hormone replacement therapy for managing menopausal symptoms, thanks to their SERM-like activity (11, 62). Quaas et al. (21) evaluated the effects of long-term isoflavone soy protein (ISP) supplementation on endometrial thickness and the incidence of endometrial hyperplasia and cancer in a population of postmenopausal women who were closely monitored by ultrasound and biopsied. The study found no significant difference in endometrial thickness between the ISP and the placebo groups. Endometrial biopsy was performed in 7.4% of ISP-treated participants and 6.8% of placebo-treated participants, with all cases in the ISP group showing benign histology. In contrast, one placebo participant developed complex endometrial hyperplasia with atypia, which progressed to Stage IB EC. Although the incidence of hyperplasia/malignancy was numerically higher in the placebo group (14.3% vs. 0%), the difference was not statistically significant (p = 0.48**).** Taken together, these findings do not indicate an increased EC risk associated with long-term ISP supplementation within the limits of this RCT. However, the small number of events and limited statistical power preclude definitive conclusions regarding long-term safety. Accordingly, these results should be interpreted with caution and cannot exclude modest risk effects. Although our systematic review focused on studies directly retrieved through the predefined search strategy, additional observational evidence on phytoestrogens and EC risk warrants consideration for contextual interpretation. A recent systematic review and meta-analysis suggested a modest inverse association between dietary isoflavone intake and EC, although results across included studies were heterogeneous and primarilybased on Asian populations with higher soy consumption (63). In a U.S. population-based case–control study, Bandera et al. found no clear association between total isoflavones or lignans and EC risk, but observed a potential protective effect restricted to lean women (BMI < 25) and for specific compounds, such as quercetin, and tofu consumption (64). More recently, a Danish case cohort analysis within the Diet, Cancer and Health study examined plasma enterolactone as a biomarker of lignan exposure and reported a suggestive but non-significant inverse trend, with no effect modification by BMI, menopausal status, or hormone therapy (65–67). These studies were not included in the present review because they did not meetthe predefined eligibility criteria. Nevertheless, their findings underscore the complexity of phytoestrogen research in EC, in which biological plausibility, population-specific dietary patterns, and exposure assessment methods may substantially influence observed associations. Overall, current evidence regarding isoflavones and EC risk remains limited and heterogeneous, and firm conclusions on long-term effects cannot be drawn in the absence of larger prospective studies with adequate event numbers and standardized exposure assessment.

### 4.8 Knowledge gaps and future research directions

Although research on the relationship between EDCs and EC has expanded in recent years, the available epidemiological evidence remains limited in both quantity and methodological robustness. Most available studies are observational and provide incomplete characterization of exposure timing, cumulative dose, and dose–response relationships, thereby limiting causal inference. This limitation has emerged consistently across the individual EDCs discussed in the present review. A major gap concerns the lack of repeated exposure measurements over time, which are necessary to account for intra-individual variability and long-term exposure patterns. Prospective studies incorporating longitudinal biospecimen collection are therefore required to improve exposure assessment and reduce misclassification. In addition, the absence of standardized analytical procedures and harmonized exposure metrics limits comparability across studies and precludes the generation of quantitative summaries. The identification and validation of reliable biomarkers of internal exposure should therefore represent a priority for future research. Another critical limitation is that existing investigations rarely evaluate the combined effects of multiple EDCs, despite human exposure occurring in real-world settings as complex mixtures. Clarifying the impact of combined EDC exposure and determining whether interactions are additive, synergistic, or antagonistic represent major challenges for future research. Understanding how such combined exposures influence estrogen-related signaling, oxidative stress, and epigenetic regulation is essential to advance the biological interpretation of epidemiological findings. Greater integration between mechanistic, epidemiological, and translational research approaches is also needed to link molecular alterations with clinical and population-level outcomes. Taken together, future studies should prioritize harmonized methodologies, rigorous control of confounding factors, and interdisciplinary collaboration to better define the role of EDCs in endometrial carcinogenesis. Such efforts are essential to support evidence-based preventive strategies and inform public health policies aimed at reducing exposure to hormonally active environmental contaminants.

### 4.9 Clinical and public health implications

Understanding the relationship between EDCs and EC has important preventive and clinical implications. At present, the available evidence does not support the implementation of individual screening or routine biomonitoring for EDC exposure in clinical practice. Although current evidence does not justify screening or biomonitoring for EDCs exposure in individual patients, awareness of environmental and occupational risk factors should be integrated into gynecologic and oncologic practice. In particular, women with known susceptibility to hormone-dependent malignancies or with metabolic and reproductive risk factors, such as obesity, polycystic ovary syndrome, or early menarche, could benefit from targeted counseling on environmental exposures.

From a preventive standpoint, lifestyle and behavioral interventions can contribute to lowering exposure levels, although their real impact remains to be demonstrated. Measures such as limiting the use of plastic containers for food storage, avoiding heating food in plastic packaging, and preferentially selecting products labeled as BPA- or phthalate-free may reduce contact with specific compounds, although the magnitude of their impact on individual cancer risk remains uncertain. Accordingly, such recommendations should be framed cautiously and interpreted within the limits of the current evidence base. At the public health and regulatory level, the data summarized in this review point to the need for strengthened environmental surveillance and more uniform regulation of industrial contaminants. Strengthening monitoring programs for EDCs residues in food and water and improving transparency in consumer product labeling may contribute to reducing population-level exposure, while further research continues to clarify causal relationships and dose-response patterns. Interdisciplinary collaboration among clinicians, toxicologists, and public health professionals remains essential to ensure that preventive strategies are grounded in robust and reproducible scientific evidence. These actions, together with improved environmental health education, may help reduce population-level exposure while further research continues to clarify causality and dose-response relationships.

### 4.10 Strengths and limitations

This systematic review provides a comprehensive analysis of the association between EDCs and EC risk, following PRISMA guidelines. A significant strength of this work lies in its transparent and reproducible methodology, including a predefined search strategy, explicit eligibility criteria, and systematic selection of studies. Several reviews have examined the association between EDCs and EC by integrating experimental and human evidence, including the review by Caserta et al. (68). In contrast, the present study was limited to human epidemiological studies and applied predefined eligibility criteria, formal risk-of-bias assessment, and a structured evaluation of exposure assessment methods and timing. The inclusion of both case-control and prospective cohort studies enabled a broader evaluation of exposure–outcome relationships across diverse epidemiological settings. In addition, mechanistic evidence from experimental models was integrated to support biological plausibility, while maintaining a clear distinction between experimental findings and population-based risk estimates. Moreover, integrating mechanistic evidence from experimental models strengthened the biological plausibility of the observed associations. The small number of eligible studies precluded quantitative synthesis and limited the ability to formally assess heterogeneity. Substantial variability in exposure assessment methods represents another important limitation. Studies based on biomonitoring approaches, such as urine or blood measurements, generally provide more objective estimates of internal exposure, whereas dietary questionnaires or self-reported data are subject to recall bias and exposure misclassification. This heterogeneity in exposure assessment may partly explain why stronger and more consistent associations were observed in biomonitoring-based studies, particularly those evaluating cadmium, compared with studies relying on dietary assessment.

Most of the included studies were observational, limiting the ability to establish causality. Consequently, reported associations should be interpreted cautiously and cannot be assumed to reflect direct causal relationships. In addition, many studies relied on single-point exposure measurements, which may not adequately capture long-term or cumulative exposure, particularly for compounds with short biological half-lives such as BPA and phthalates. This limitation may have resulted in exposure misclassification and attenuation of true associations.

Finally, incomplete adjustment for relevant confounders, including body mass index, dietary factors, and hormone therapy use, together with limited sample sizes and absence of subgroup analyses, further constrained the interpretation of the findings. These limitations highlight the need for larger, well-designed prospective studies with standardized exposure assessment and comprehensive confounder control to clarify the role of EDCs in endometrial carcinogenesis.

## 5 Conclusions

In conclusion, current epidemiological evidence suggests a potential association between cadmium exposure and increased EC risk, whereas data for other EDCs remain inconclusive. The substantial heterogeneity in exposure assessment, study design, and confounder adjustment prevents definitive conclusions. Well-designed longitudinal studies with standardized and repeated exposure measurements are necessary to elucidate the mechanistic and causal relationships between EDCs exposure and endometrial carcinogenesis, ultimately informing preventive strategies against hormone-related environmental risks. Key aspects, such as timing of exposure and cumulative dose, are seldom addressed, despite their potential relevance. Most available studies are observational, involve small sample sizes, and rely on single-point biomarker assessments, limiting the ability to draw firm causal conclusions. Well-designed longitudinal studies with standardized and repeated exposure measurements are necessary to elucidate the mechanistic and causal relationships between EDC exposure and endometrial carcinogenesis, ultimately informing preventive strategies against hormone-related environmental risks.

## Data Availability

The datasets presented in this study can be found in online repositories. The names of the repository/repositories and accession number(s) can be found below: https://inplasy.com/inplasy-2025-6-0030/.
